# Which Is Better in Clinical and Radiological Outcomes for Lumbar Degenerative Disease of Two Segments: MIS-TLIF or OPEN-TLIF?

**DOI:** 10.3390/jpm12121977

**Published:** 2022-11-30

**Authors:** Weiran Hu, Guang Yang, Hongqiang Wang, Xiaonan Wu, Haohao Ma, Kai Zhang, Yanzheng Gao

**Affiliations:** 1 Department of Spine and Spinal Cord Surgery, People’s Hospital of Zhengzhou University, Zhengzhou 450003, China; 2 Department of Spine and Spinal Cord Surgery, People’s Hospital of Henan University, Zhengzhou 450003, China

**Keywords:** TLIF, MIS-TLIF, lumbar degenerative disease, clinical outcome

## Abstract

Objective: To compare the clinical and radiological outcomes of minimally invasive transforaminal lumbar interbody fusion (MIS-TLIF) and traditional open transforaminal lumbar interbody fusion (OPEN-TLIF) in the treatment of two-level lumbar degenerative diseases. Methods: The clinical data of 112 patients were retrospectively analyzed, and were divided into an MIS-TLIF group and OPEN-TLIF group. The operative time, intraoperative fluoroscopy, blood loss, postoperative drainage volume, bed rest time, the content of creatine kinase(CK) and complications, were recorded. VAS score and ODI index were used to evaluate clinical efficacy. Bridwell grading was used to evaluate postoperative interbody fusion. Screw position was evaluated by Rao grading. Results: Compared with the OPEN-TLIF group, the MIS-TLIF group had longer operation times, more intraoperative fluoroscopy times, but shorter postoperative bed times (*p* < 0.05). There were no significant differences in blood loss, postoperative drainage and postoperative CK content between the two groups (*p* > 0.05). There was no difference in VAS score and ODI index during the follow-up (*p* > 0.05). There was no significant difference in the interbody fusion rate between the two groups (*p* > 0.05). There was no significant difference in the distribution of type A screws, but the type B screw in the MIS-TLIF group was higher (*p* < 0.05). There was no difference in the incidence of complications between the two groups (*p* > 0.05). Conclusion: The postoperative quality of life score and radiological outcomes of the two types of surgery in two-level lumbar degenerative diseases was similar, and there was no significant difference in muscle injury and complications, but the operation time and intraoperative radiation exposurewere higher than in the OPEN-TLIF group, and the pedicle screws were more likely to deviate laterally out of the vertebral body. Therefore, OPEN-TLIF is recommended for patients with lumbar degenerative diseases of two segments.

## 1. Introduction

Lumbar degenerative disease is a common disease in spinal surgery that mostly occurs in the elderly, with main clinical manifestations such as sciatica, low back pain, and cauda equina syndrome. Recently, transforaminal lumbar interbody fusion (TLIF) has become a common surgical approach for the clinical treatment of lumbar degenerative disease [1]. The advantage of TLIF is that the nerve is exposed laterally by removing part of the facet joint, and the traction of the nerve root is reduced to avoid potential nerve injury. TLIF preserves the integrity of the posterior column by reducing the removal of the spinous process. OPEN-TLIF has been a safe and effective lumbar fusion procedure [2]. A systematic review of 192 studies concluded that OPEN-TLIF has advantages over PLIF in complication rate, blood loss, and operation duration. The clinical outcome is similar, with a slightly lower postoperative ODI score for TLIF [3]. However, extensive dissection of the paraspinal muscles is required in the MIS-TLIF procedure, and this may result in postoperative paravertebral muscle atrophy and low back pain [4]

With the progress of spinal surgery technology and the development of minimally invasive surgical techniques, Foley et al. [5] proposed in 2002 to complete TLIF surgery under an expandable tube via the wistle approach, and named this minimally invasive transforaminal lumbar interbody fusion (MIS-TLIF). The decompression and fusion procedure can be operated between the multifidus muscle and longissimus muscle to reduce the injury of paraspinal muscles and soft tissues [6]. Previous studies concluded that there was no significant difference in postoperative quality of life score between Open-TLIF and MIS-TLIF [7,8], and some scholars believed that MIS-TLIF was superior to OPEN-TLIF in intraoperative blood loss and bedridden time [9,10]. However, almost all the conclusions were based on single-segment lumbar degenerative diseases [9,10,11,12,13], and the merits of the two surgical methods in two-segment lumbar diseases are still controversial.

With the aging of the population, patients with two segments lumbar degenerative diseases are not uncommon. According to a long-term follow-up study, about 20% of patients with lumbar degenerative diseases have two or more levels of lumbar disc herniation [14]. For these patients, multilevel surgery may be necessary, and choosing a more appropriate surgical procedure will reduce surgical trauma. Whether MIS-TLIF still has advantages for two segments degenerative lumbar diseases is still debatable. To our knowledge, there has been no literature focused on the comparison of the two surgical methods for two segments disorders. In clinical practice, we found that the two surgical methods have similar efficacy. In order to verify this hypothesis, a total of 112 patients with lumbar degenerative diseases of two segments treated in our institution from January 2015 to September 2021 were included in this study. MIS-TLIF and Open-TLIF were used for surgical treatment, and we compared the clinical and radiological outcomes of the two methods.

## 2. Materials and Methods

### 2.1. Inclusion and Exclusion Criteria

Inclusion criteria: (1) patients with typical clinical manifestations of lumbar spinal stenosis, lower extremity neurological symptoms, low back and leg pain and failed to respond to standard conservative treatment for 3 months; (2) lumbar imaging examination showed spinal stenosis with or without lumbar spondylolisthesis; (3) CT and MRI confirmed two-level disc degeneration, the abnormal changes in imaging was consistent with clinical symptoms.

Exclusion criteria: (1) deformity or combined with grade III or above lumbar spondylolisthesis; (2) lumbar infection, tumor, severe osteoporosis or motor neuron disease; (3) patients who had undergone lumbar surgery or local block therapy; (4) incomplete imaging data and loss of follow-up.

### 2.2. General Information of Patients

A total of 112 patients with lumbar degenerative diseases of two segments were included according to the inclusion and exclusion criteria. There were 60 patients in the OPEN-TLIF group, which was also treated as the control, including 34 males and 26 females. There were 52 patients in MIS-TLIF group, including 28 males and 24 females. There was no significant difference in preoperative general information between the two groups (Table 1). All patients were treated by experienced physicians using the Quadrant minimally invasive operating system (Beijing Fule Technology Development Co., Ltd., Beijing, China). This study was reviewed and approved by the Ethics Committee of Henan Provincial People’s Hospital(IRB approval number 2021–173). All patients signed informed consent.

### 2.3. Surgical Method of OPEN-TLIF

After general anesthesia, patients in the Open-TLIF group were placed in a prone position, and the operation area was routinely disinfected. The paravertebral muscle was separated from the spinous process, lamina, and facet capsule. The facet joints were exposed, three pedicle screws were inserted. The facet joint was bitten, the lamina was removed, the hyperplastic ligamentum flavum was removed, the dural sac and nerve roots were exposed, the protruding nucleus pulposus was removed, the intervertebral disc tissue was cleaned, the upper and lower cartilaginous endplates were scraped, the trimmed bone particles were implanted in the intervertebral space, and the cage was implanted in an oblique way. Whether to perform contralateral decompression was determined according to the preoperative symptoms and imaging manifestations. A paravertebral drainage tube was implanted, and the incision was sutured layer by layer.

### 2.4. Surgical Method of MIS-TLIF

The anesthesia mode and intraoperative position of patients in MIS-TLIF group were consistent with those in the OPEN-TLIF group. A posterior median incision was made, the subcutaneous skin was separated, and the skin was separated to the fascia layer on both sides. The fascia was cut 2 cm beside the midline at the intermuscular space between the longissimus muscle and multifidus muscle. A guide wire was implanted in the muscle space by blunt separation. After confirming the correct location, the quadrant channel was inserted. The remaining surgical procedures were the same as OPEN-TLIF surgery.

### 2.5. Postoperative Management

Prophylactic antibiotics were applied for 72 h after operation, and tower limb activity and symptom relief of patients were observed regularly. Braces were worn regularly for 3 months. Follow-up was performed at 1 week, 6 and 12 months after operation. X-ray and CT examinations were performed.

### 2.6. Observational Index

We used VAS score and ODI index to evaluate the clinical efficacy after the surgery. VAS score for low back pain and VAS score for leg pain and ODI index were recorded before operation, 1 week, 3 months and 12 months after operation. 

Muscle injury during the perioperative period was evaluated according to the changes of blood creatine kinase (CK), and the CK content of the two groups was measured at preoperative, 3 days and 1 week after operation.

Postoperative intervertebral fusion was evaluated by the grading of lumbar fusion proposed by Bridwell et al. [15]. Grade 1 showed complete fusion of the intervertebral space with trabecular reconstruction, and the grade 2 showed incomplete fusion of the intervertebral space with trabecular reconstruction.

**Figure 1:**
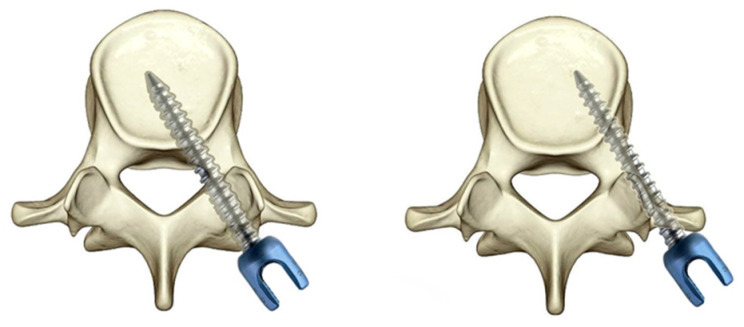
Diagram of type A and B screw according to RAO grading.

The screw position was evaluated by the classification proposed by Rao et al. [16]. Type A0 means that the screw did not penetrate the medial wall of the pedicle. Type A1 means that the screw penetrated the medial wall of the pedicle less than 2 mm. Type A2 means that the screw penetration of the medial wall of the pedicle was greater than 2 mm and less than 4 mm. Type B0 means that the screw did not penetrate the lateral wall of the pedicle. Type B1 means that the screw penetrated the lateral wall of the pedicle less than 2 mm. Type B2 means that the screw penetrated the lateral wall of the pedicle more than 2 mm and less than 4 mm.(Figure 1).

### 2.7. Statistical Analysis

SPSS 22.0(SPSS Inc., Chicago, IL, USA) was used for comparison and analysis of all data. Counting data were expressed as (X ± s). Normality of the data distribution was confirmed by Shapiro–Wilk testing. An independent sample T-test was used for comparison of the operation time, fluoroscopy, the amount of bleeding, the volume of drainage and the bed rest time between the two groups. An independent sample T-test was also used to compare the quality-of-life scores at each follow-up point between the two groups. Measurement data are expressed as (n%) and the Chi-square test was used for comparison of the general information of the patients, the complications after surgery and the distribution of screw types. Effect size (Cohen d) was calculated to examine the effect of statistical differences, and was classified as weak (≤0.49), moderate (0.5–0.79), or large (≥0.8). *p* < 0.05 was considered significant.

## 3. Results

### 3.1. Surgery Related Information

The patients were followed up for 14.7 ± 2.1 months. There were no significant differences in general information between the two groups (Table 1). Compared with the OPEN-TLIF group, the MIS-TLIF group had longer operation time, more intraoperative fluoroscopy times, but shorter postoperative bed rest time; the differences were statistically significant (*p* < 0.05). There was no significant difference in intraoperative blood loss, postoperative drainage volume (*p* > 0.05) (Table 2). 

### 3.2. CK Perioperative Content

**Figure 2:**
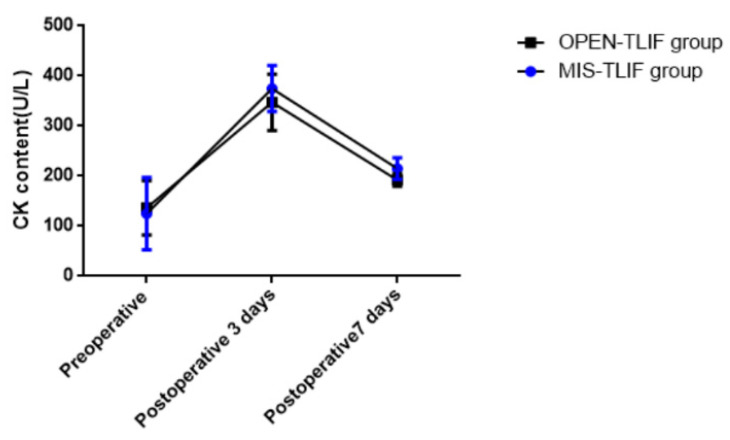
Changes of perioperative CK content in MIS-TLIF and Open-TLIF groups.

CK content was measured between the two groups at 3 and 7 days postoperatively. In both groups we found that the CK increased in the 3 days postoperatively, but the CK reached a significant decrease in the 7 days postoperatively. When we compared the CK content between the two groups, no significant differences were found at 3 and 7 days postoperatively(Figure 2). 

### 3.3. The Change of VAS Score and ODI Index

VAS score and ODI index was used to evaluate the clinical effect after operation. The VAS score and ODI index in the two groups were significantly improved one week after operation compared with those before operation (*p* < 0.05), and the clinical efficacy showed a trend of further improvement with the extension of follow-up time. The VAS score of low back pain in the MIS-TLIF group was lower than that in the OPEN-TLIF group 1 week after operation (*p* < 0.05), but there was no significant difference at 3 and 12 months after operation (*p* > 0.05). There was no significant difference in ODI index between the two groups at each node during follow-up (*p* > 0.05) (Table 3, Table 4 and Table 5).

### 3.4. The Screws Classified by Rao Grading

A total of 672 pedicle screws were inserted in the two groups. In the MIS-TLIF group, 360 screws were implanted, including 302 A0 screws, 46 A1 screws and 12 A2 screws. There were 214 B0 screws, 82 B1 screws and 64 B2 screws. In the OPEN-TLIF group, 312 screws were implanted, including 228 A0 screws, 54 A1 screws and 5 A2 screws. There were 242 B0 screws, 62 B1 screws and 8 B2 screws. There was no significant difference in the distribution of type A screws between the two groups (*p* > 0.05). There were significant differences in the distribution of type B screws between groups. This means that screw penetration of the lateral wall of the pedicle was more likely in the MIS-TLIF group (*p* < 0.05) (Table 6.)

### 3.5. Fusion Level by Bridwell Grading

According to the Bridwell classification, 30 cases (57.7%) of grade 1 fusion and 22 cases (42.3%) of grade 2 fusion in the MIS-TLIF group. In the OPEN-TLIF group, 34 cases (56.7%) had grade 1 fusion and 26 cases (43.3%) had grade 2 fusion. There was no significant difference in interbody fusion rate between the two groups (*p* > 0.05).

### 3.6. Complications

There were no complications such as internal fixation loosening and displacement in the two groups. There were six cases of dural tear in the MIS-TLIF group and two cases in the OPEN-TLIF group, and these patients were treated with pressure dressing of the surgical incision; the drainage was removed with a delay of 1 week after operation. There were six cases in the MIS-TLIF group, and four cases in the OPEN-TLIF group experienced numbness of lower extremities; their symptoms were relieved after symptomatic treatment. There were two cases of poor wound healing in the OPEN-TLIF group, and these patients were treated with debridement and suturing. There was no significant difference in the incidence of complications between the two groups (*p* > 0.05) (Table 2).

## 4. Discussion

**Figure 3:**
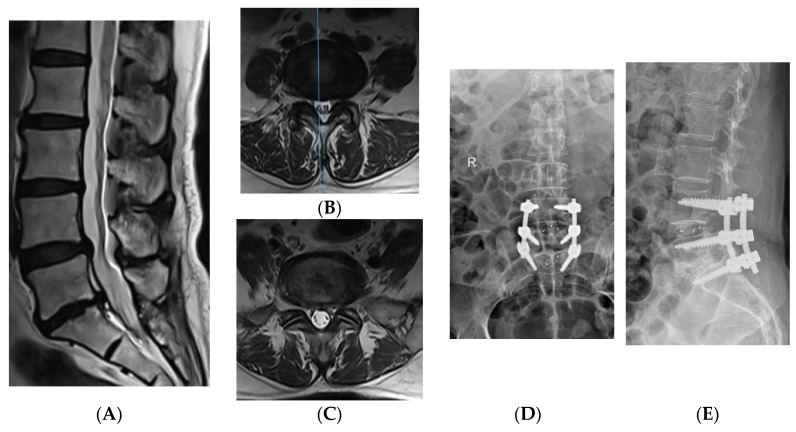

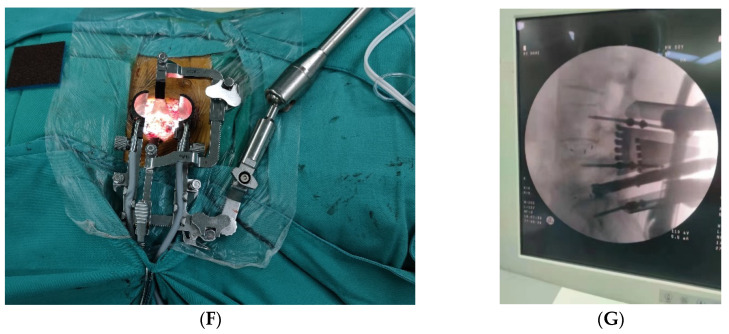
A typical case in the MIS-TLIF group. A 64-year-old woman was admitted to the hospital because of “waist pain for more than 10 years, aggravated with lower limb discomfort for 3 months”, and underwent two-stage Mis-TLIF surgery. (**A**–**C**) show MRI imaging examination of disc herniation in L4/5 and L5/S1, disc degeneration in L5/S1, and narrowing of intervertebral space. (**D**,**E**) show postoperative X-ray. (**F**,**G**) The Quadrant minimally invasive operating system and an intraoperative fluoroscopic image, respectively.

The clinical efficacy of OPEN-TLIF were verified [17,18]. Kunder et al. [17] compared the advantages and disadvantages of PLIF and OPEN-TLIF in a meta-analysis, and the results showed that OPEN-TLIF was superior to PLIF in terms of operation time, intraoperative blood loss, and incidence of complications. MIS-TLIF adopts the paravertebral space approach to complete decompression and intervertebral bone fusion through an expandable channel, which can preserve the integrity of paravertebral muscles and posterior structures of the vertebral body [19,20]. However, the channel limits the operation space, experienced surgical techniques and necessary surgical tools are required, and the learning curve is steep [21]. In this study, we found that the postoperative quality of life score and radiological outcomes of the two methods was similar but the operation time and intraoperative radiation exposure were higher in the MIS-TLIF group. A typical case is shown in Figure 3.

During follow-up we found that the VAS score of low back pain in the MIS-TLIF group was lower than that in the OPEN-TLIF group 1 week after operation (*p* < 0.05); this was the only difference in the quality-of-life score between the two groups. The VAS score of low back pain and leg pain and ODI index had no difference in other follow-up nodes. This means that the MIS-TLIF group was superior to the OPEN-TLIF group only in early relief of low back pain, but the clinical efficacy of the two groups was similar over time. Modi and Berkman demonstrated that both techniques have the similar efficacy in single-stage lumbar degenerative disease. Our study demonstrated that the efficacy was comparable when the indications for surgery were expanded to include two-level lumbar degenerative disease [22,23].

We found that the MIS-TLIF group had higher operation time and fluoroscopy times. The increase of fluoroscopy prolonged the operation time and increased radiation exposure to doctors and patients. A number of studies have also confirmed this conclusion [24,25]. Arif et al. [26] found that the operation time of MIS-TLIF was increased by 126.3 min and fluoroscopy time was increased by 22.9 s compared with the traditional method. By monitoring the exposure dose, related research found that the radiation dose of MIS-TLIF surgery was 30 µSV higher than conventional surgery.

CK levels measured at 3 and 7 days after surgery were similar in the two groups. Muscle injury results in an increase in cell membrane passage, which leads to the release of CK into the bloodstream. Continuous monitoring of CK after surgery showed that there was no significant difference in muscle injury between the two groups. MIS-TLIF surgery is designed to minimize muscle irritation, but our study found that when MIS-TLIF was extended to 2-level surgery, the results of muscle injury did not improve compared with OPEN-TLIF.

According to Bridwell classification, there was no significant difference in the postoperative interbody fusion rate between the two groups (*p* < 0.05). This means the surgery method did not affect the fusion rate. Kang et al. [18] found that there was no significant difference in interbody fusion rate between MIS-TLIF (RR = 2.13, 95%CI: 1.39–3.27) and OPEN-TLIF (RR = 2.13, 95%CI: 1.39–3.27). This is consistent with the results of our study. Kim et al. [13] conducted a 5-year follow-up study on the fusion results of MIS-TLIF, and the authors found that the fusion rate was 97.7%. All these studies showed that the postoperative fusion rate could achieve satisfactory results whether open or minimally invasive.

In this study, we found no significant difference in the incidence of complications between the two groups. However, concerning dural tear, we found the incidence of dural tear was higher in the MIS-TLIF group. Dural tears occurred in six cases in the MIS-TLIF group and only two cases in the OPEN-TLIF group. The reason may be that the learning curve of MIS-TLIF is steep, and the operation space is narrow due to the limitation of the channel. Lee et al. [21] conducted a large sample size study to evaluate the learning curve of MIS-TLIF. The authors found that only after at least 44 cases of surgery could surgeons truly master the skill, shorten the operation time and reduce the amount of fluoroscopy, and patients could obtain satisfactory clinical efficacy. It is also worth noting that serious complications have occurred due to the surgeon’s familiarity with MIS operations: duodenal rupture occurred in one case and cage loosening occurred in two cases. A study by Kang et al. [27] counted dural tears in the MIS-TLIF surgery, which occurred in one out of four patients undergoing primary surgery, and in four out of nineteen patients undergoing revision surgery. Goertz et al. [28] found that the incidence of dural tears was higher in obese patients after MIS-TLF surgery. The results of the above studies indicate that the indications for MIS-TLIF surgery should be strictly controlled, and appropriate cases should be selected to improve the safety of surgery.

In this study, there were no complications due to pedicle screw misplacement in either group. However, when the study focused on the accuracy of screw placement, and the position of the screw was evaluated according to Rao classification, the number of B-type screws in the MIS-TLIF group was higher than that in the OPEN-TLIF group (*p* < 0.05). More type B screws means that more screws broke through the lateral wall in the MIS-TLIF group. Screw penetration of the lateral wall limits screw length, reduces holding force, and may increase surgical complications. The reason may be that MIS-TLIF is performed using Quadrant channels and the narrow space may limit the angle and position of pedicle screw implantation. Previous studies have reported revision of MIS-TLIF due to postoperative neurological symptoms caused by screw misplacement. Venier et al. [29] analyzed the use of CT navigation to guide pedicle screw placement in MIS-TLIF, and the results showed that the screw placement accuracy was 95.3%, and 19 screws (4.7%) deviated into the spinal canal. The authors believed that the narrow space under the channel limited the accuracy of screw placement. The study of Zhao et al. [30] found that the injury rate of the upper facet during MIS-TLIF was 34.07% (62/182). After logistics regression analysis, the author found that a body mass index over 30 kg/m^2^ and L5 pedicle screw placement were independent risk factors for facet joint injury.

This study has limitations. First, the number of cases was limited. The incidence of lumbar degenerative diseases of two segments was lower, and the lack of follow-up data for some patients further limited the sample size. Second, the follow-up time was limited. Although all the patients achieved fusion in the follow-up time of this study, the difference in long-term efficacy between the two surgical methods still needs to be observed, and the difference in long-term complications between the two surgical methods still needs to be followed up. Third, this study was a single center retrospective study. The advantages and disadvantages of the two methods should be further compared in future studies with larger sample sizes, longer follow-ups and in multiple centers.

## 5. Conclusions

In conclusion, the postoperative quality-of-life score and radiological outcomes of the two types of surgery in two-level lumbar degenerative diseases is similar, and there is no significant difference in muscle injury and complications. However, the operation time and intraoperative radiation exposure are higher than those of the OPEN-TLIF group, and the pedicle screws are more likely to deviate laterally out of the vertebral body. Therefore, OPEN-TLIF is recommended for patients with lumbar degenerative diseases of two segments.

## Figures and Tables

**Table 1:** General information of the two groups.

	Mis-TLIF Group (*n* = 52)	Open-TLIF Group (*n* = 60)	*p* Value
Age (years)	64.18 ± 8.17	66.24 ± 7.16	0.271
Gender (Male/Female)	28/24	34/26	0.322
BMI (kg/m^2^)	32.41 ± 3.87	33.74 ± 4.15	0.642
Lumbar spondylolisthesis	18(18/52)	14(14/60)	0.196
Operative site			0.147
L3/4 and L4/5	16	22	
L4/5 and L5/S1	36	38	

**Table 2:** Surgery-related indicators and complications of the two groups.

	Mis-TLIF Group (*n* = 52)	Open-TLIF Group (*n* = 60)	Cohen d	*p* Value
Operation time (mins)	287.74 ± 32.17	232.96 ± 42.56	1.45	**0.014**
Fluoroscopy	12.74 ± 2.35	7.56 ± 3.21	1.84	**0.032**
Amount of bleeding (mL)	537.62 ± 112.78	574.97 ± 134.26	−0.30	0.184
Volume of drainage (mL)	372.86 ± 165.41	354.91 ± 143.92	0.12	0.081
Bed rest time (days)	4.50 ± 1.08	6.24 ± 1.34	−1.43	**0.037**
Complications				0.792
CSF leak	6	2		
Poor wound healing	0	2		
Numbness	6	4		

**Table 3:** Changes in low back pain VAS score during the follow-up.

	Low Back Pain VAS Score			
	Pre-Operation	Postoperative 1 Week	Postoperative 3 Months	Postoperative 12 Months
MIS-TLIF	5.78 ± 2.12	2.03 ± 1.43 a	1.69 ± 0.72	1.19 ± 0.75
OPEN-TLIF	5.22 ± 3.37	3.11 ± 1.04	1.42 ± 0.46	1.08 ± 0.82
Cohen d	0.20	−0.86	0.45	0.14
*p* value	0.432	**0.012**	0.067	0.081

**Table 4:** Changes in leg pain VAS score during the follow-up.

	Leg Pain VAS Score			
	Pre-Operation	Postoperative 1 Week	Postoperative 3 Months	Postoperative 12 Months
MIS-TLIF	7.35 ± 1.15	2.46 ± 0.75	1.34 ± 0.87	1.04 ± 0.59
OPEN-TLIF	8.04 ± 2.01	2.45 ± 1.25	1.67 ± 0.43	1.15 ± 0.64
Cohen d	−0.42	0.01	−0.48	−0.18
*p* value	0.134	0.237	0.073	0.126

**Table 5:** Changes in ODI index during the follow-up.

	ODI (%)			
	Pre-Operation	Postoperative 1 Week	Postoperative 3 Months	Postoperative 12 Months
MIS-TLIF	76.17 ± 6.63	13.71 ± 2.38	12.26 ± 3.54	12.46 ± 4.31
OPEN-TLIF	68.42 ± 7.47	16.24 ± 3.12	15.17 ± 2.46	14.14 ± 3.37
Cohen d	1.11	−0.91	−0.95	−0.43
*p* value	0.243	0.176	0.102	0.065

**Table 6:** Distribution of screw types of the two groups according to RAO grading.

	OPEN-TLIF Group (*n* = 360)	MIS-TLIF Group (*n* = 312)	*p* Value
A type screw			0.312
A0 screw	151	124	
A1 screw	23	27	
A2 screw	6	5	
B type screw			0.021
B0 screw	107	121	
B1 screw	41	31	
B2 screw	32	4	

## Data Availability

All data are described in the manuscript. The datasets used and/or analyzed in the present study are available from the corresponding author upon reasonable request.
